# Beyond the Bedside: Proactive Simulation to Safeguard against Behavioral Events in Pediatric Emergency Departments

**DOI:** 10.1097/pq9.0000000000000832

**Published:** 2025-08-29

**Authors:** Diana Gomez, Janice Lee Yanez, Tianna Schiappa Pietra

**Affiliations:** From the Patient Safety, Nicklaus Children’s Hospital, Miami, Fla.

## Introduction:

Behavioral health events in pediatric acute care settings, particularly in emergency departments (EDs), are increasingly recognized as critical incidents that can significantly impact patient safety, staff well-being, and the overall efficiency of healthcare delivery. These events often involve unpredictable behaviors that require rapid, coordinated responses from multidisciplinary teams. In such high-stress environments, the ability of staff to respond effectively is closely tied to their confidence, training, and ability to work collaboratively. Simulation-based training in controlled, realistic environments has emerged as a powerful tool to enhance workforce preparedness, build resilience, and foster a culture of safety.^1–3^

## Methods:

The primary objective of this initiative was to proactively prepare ED staff to manage pediatric behavioral health crises through interdisciplinary simulation-based training. By replicating real-world scenarios, the simulation aimed to improve staff confidence, enhance communication and teamwork, and promote a proactive approach to patient and staff safety. The broader goal was to embed a culture of responsiveness and collaboration in managing behavioral emergencies, ultimately improving outcomes for both patients and healthcare providers. The Patient Safety Team at Nicklaus Children’s Hospital partnered with ED leadership to design and implement a proactive simulation initiative. A multidisciplinary planning team was assembled, including nurses, physicians, pharmacists, security personnel, and child life specialists. Together, they developed a realistic simulation scenario grounded in actual patient safety events. The scenario was designed to test clinical decision-making, communication, and crisis management skills. To further enhance the authenticity of the simulation, input was sought from the hospital’s Family Advisory Council, ensuring that the patient and family perspective was meaningfully integrated into the training.^4,5^

## Results:

Following the simulation, participants completed a structured survey to assess the impact of the training. Results were overwhelmingly positive: 98% of participants reported increased confidence in managing similar real-life behavioral events, and 100% believed that the simulation would contribute to improved patient outcomes (see Table 1). Participants highlighted the value of interdisciplinary collaboration, noting that the exercise improved communication, clarified roles, and strengthened team dynamics. Many also expressed appreciation for the opportunity to practice in a safe environment, which allowed them to reflect on their responses and identify areas for improvement.

**Table 1.** Summary of Simulation Results

Metric	Result, %	Notes
Participant confidence postsimulation	98	Participants reported increased confidence in managing behavioral events
Perceived impact on patient outcomes	100	All participants believed the simulation would improve patient outcomes

The table summarizes the key outcomes from the proactive simulation training conducted at Nicklaus Children’s Hospital. The results reflect participant feedback on confidence levels and perceived impact on patient outcomes following the simulation.

## Conclusions:

This proactive simulation initiative demonstrated the effectiveness of interdisciplinary, scenario-based training in preparing staff for pediatric behavioral health events in the ED. By fostering collaboration, enhancing confidence, and promoting a culture of safety, the simulation contributed to improved readiness and resilience among healthcare teams. The success of this initiative underscores the importance of integrating proactive safety simulations into routine training programs across healthcare organizations. Future efforts will focus on expanding simulation opportunities to additional departments and disciplines, ensuring that all staff are equipped to respond effectively to behavioral health crises and uphold the highest standards of patient and staff safety.^1–5^

## References

[1] Thim S Henriksen TB Laursen H. Simulation-based emergency team training in pediatrics: a systematic review. Pediatrics. 2022;149:e2021054305. 35237809 10.1542/peds.2021-054305

[2] Kim E Song S Kim S. Development of pediatric simulation-based education—a systematic review. BMC Nurs. 2023;22:291. 37641090 10.1186/s12912-023-01458-8 PMC10463597

[3] Saeed S Hegazy NN Malik MGR. Transforming the delivery of care from “I” to “we” by developing crisis resource management skills in pediatric interprofessional teams through simulation. BMC Med Educ. 2024;24:649. 38862911 10.1186/s12909-024-05459-2 PMC11167930

[4] Saidinejad M Duffy S Wallin D; American Academy of Pediatrics Committee on Pediatric Emergency Medicine. The management of children and youth with pediatric mental and behavioral health emergencies. Pediatrics. 2023;152:e2023063256.37584106

[5] Laprime A Kanaley R Keller A. Improving employee safety through a comprehensive patient behavioral program. Hosp Pediatr. 2024;14:356–363. 38606483 10.1542/hpeds.2023-007714

